# Forehead electrodes sufficiently detect propofol-induced slow waves for the assessment of brain function after cardiac arrest

**DOI:** 10.1007/s10877-019-00282-3

**Published:** 2019-02-20

**Authors:** Jukka Kortelainen, Eero Väyrynen, Ilkka Juuso, Jouko Laurila, Juha Koskenkari, Tero Ala-Kokko

**Affiliations:** 1 Physiological Signal Analysis Team, Center for Machine Vision and Signal Analysis, MRC Oulu, University of Oulu, P.O. Box 4500, 90014 Oulu, Finland; 2 Cerenion Oy, Elektroniikkatie 3, 90590 Oulu, Finland; 3 Research Group of Surgery, Anaesthesiology and Intensive Care, Medical Faculty, University of Oulu, P.O. Box 5000, 90014 Oulu, Finland; 4 Division of Intensive Care Medicine, MRC Oulu, University of Oulu and Oulu University Hospital, P.O. Box 21, 90029 Oulu, Finland

**Keywords:** Anesthesia, Brain injury, Electroencephalogram, Intensive care, Monitoring, Propofol

## Abstract

In a recent study, we proposed a novel method to evaluate hypoxic ischemic encephalopathy (HIE) by assessing propofol-induced changes in the 19-channel electroencephalogram (EEG). The study suggested that patients with HIE are unable to generate EEG slow waves during propofol anesthesia 48 h after cardiac arrest (CA). Since a low number of electrodes would make the method clinically more practical, we now investigated whether our results received with a full EEG cap could be reproduced using only forehead electrodes. Experimental data from comatose post-CA patients (N = 10) were used. EEG was recorded approximately 48 h after CA using 19-channel EEG cap during a controlled propofol exposure. The slow wave activity was calculated separately for all electrodes and four forehead electrodes (Fp1, Fp2, F7, and F8) by determining the low-frequency (< 1 Hz) power of the EEG. HIE was defined by following the patients’ recovery for six months. In patients without HIE (N = 6), propofol substantially increased (244 ± 91%, mean ± SD) the slow wave activity in forehead electrodes, whereas the patients with HIE (N = 4) were unable to produce such activity. The results received with forehead electrodes were similar to those of the full EEG cap. With the experimental pilot study data, the forehead electrodes were as capable as the full EEG cap in capturing the effect of HIE on propofol-induced slow wave activity. The finding offers potential in developing a clinically practical method for the early detection of HIE.

## Introduction

A substantial portion of comatose patients admitted to intensive care following successful resuscitation after cardiac arrest (CA) suffers from hypoxic ischemic encephalopathy (HIE) [1]. Early detection of HIE and accurate prognosis is challenging during the first days as reliable neurological examination is hindered by sedation and therapeutic hypothermia [2]. To improve prognostic accuracy, clinical examination has been supported with additional methods, consisting of electrophysiological measurements, blood biomarkers, and brain imaging [3]. While known to hold prognostic information in HIE for decades, the recent progress in measurement devices, including improved wireless and computational properties, has made electroencephalogram (EEG) possibly the most promising clinical tool for the assessment of brain function after CA. This is also supported by the findings indicating the usefulness of simplified EEG-derived parameters in the early detection of HIE [4–6].

We have recently proposed a novel method to evaluate HIE by assessing EEG slow wave (< 1 Hz) activity [6]. Slow waves are the most important EEG signatures of non-rapid eye movement sleep and they are also seen during propofol anesthesia [7–9]. Playing an important role in several neurophysiological phenomena related to higher cognitive function [10, 11] slow waves are considered to have central role in mediating consciousness [12]. We investigated in an experimental pilot study whether EEG slow waves hold prognostic information of HIE in comatose patients admitted to intensive care after CA. Our findings suggested that the lack of propofol-induced slow wave activity 48 h after CA refers to poor outcome.

In the demanding and busy intensive care environment, practicality of the measurement setup is highly valued. For successful EEG recording, one of the key issues is the electrodes, which should be easy to attach and should maintain their contact securely, thus enabling long-term high-quality signal acquisition. Electrodes attachable to the patient’s forehead with minimal preparation offer potentially the most convenient approach for EEG recording in the intensive care unit (ICU) where patients usually lie on their back. However, limiting the number of electrodes to the forehead area potentially jeopardizes the reliable interpretation of the signal. In this study, we investigated whether the slow wave activity during propofol anesthesia after CA, previously shown to differentiate patients with a good and with a poor outcome, could be detected using only forehead electrodes instead of the full 19-channel EEG cap.

## Materials and methods

**Fig. 1:**
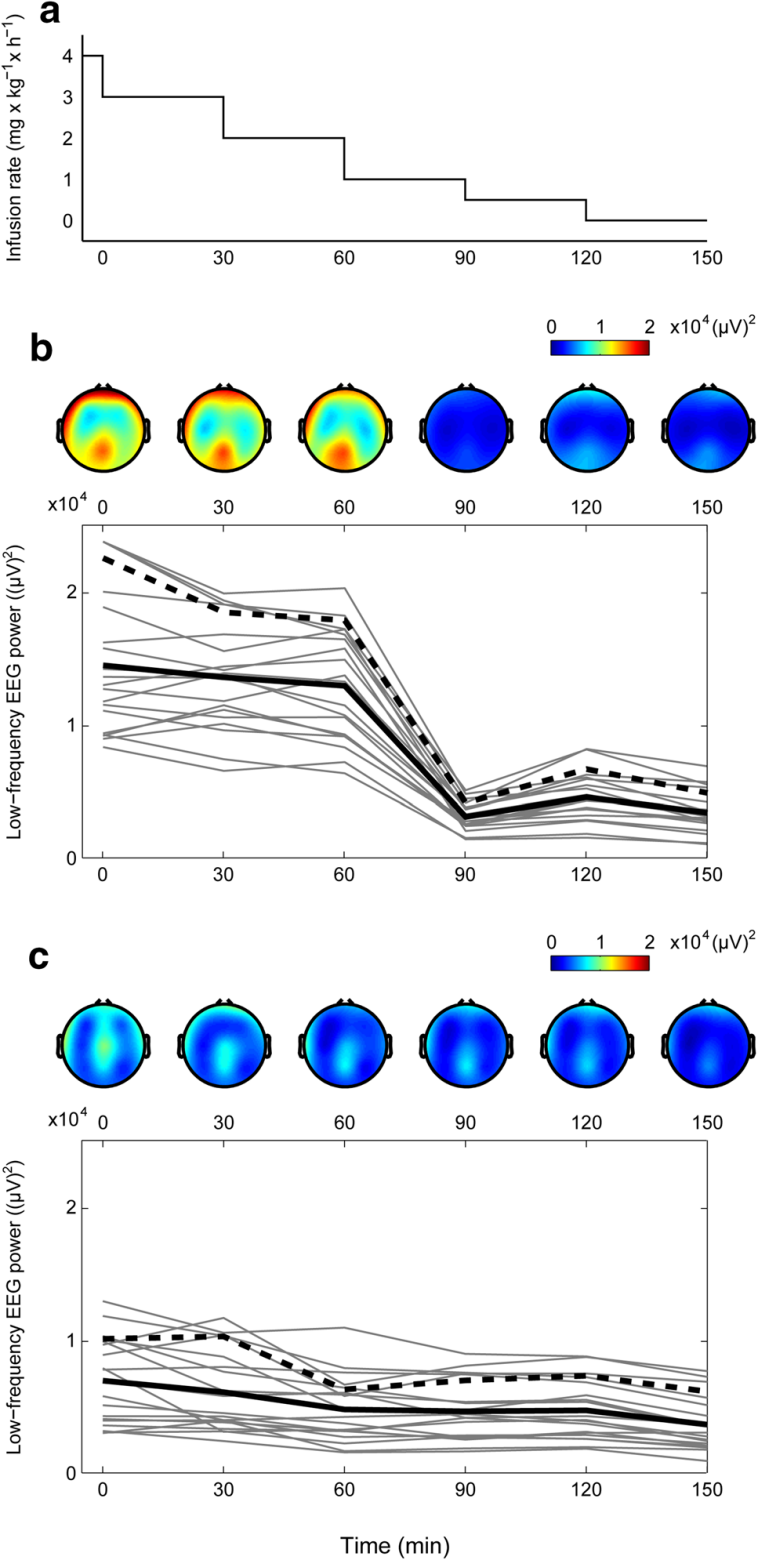
Effect of propofol on EEG slow wave activity in four forehead channels and all 19 channels of full EEG cap. **a** Propofol infusion rate during the experiment. **b** Low-frequency (< 1 Hz) EEG power representing the slow wave activity of a patient with good neurological outcome. The average low-frequency power (thick curve) is calculated from all 19 single channel powers (gray curves). The average power of the four forehead electrodes (dashed curve) is also shown. The topographic distribution of the low-frequency EEG power at different phases of the experiment is given above the curves. **c** The same data of a patient with poor neurological outcome

In the study, previously collected pilot data were used. A detailed description of the clinical protocol has been given in our previous publication [6]. In short, ten comatose patients resuscitated from out-of-hospital CA were included in the study. The patients went through an experiment in which their EEG was recorded approximately 48 h after CA while the infusion of propofol, used for sedation of the patient, was incrementally decreased to zero. The decrease was started from the highest acceptable infusion rate during the intensive care (4 mg kg^−1^ h^−1^) and continued following a predefined protocol (see Fig. 1a) until the drug administration was finally switched off. EEG was recorded using a 19-channel cap with electrodes located according to the 10/20 international system. A sampling frequency of 500 Hz and bandwidth of 0.053–125 Hz was used and the signals were referenced to common average. After the experiment, the patients were followed for 6 months after which their neurological outcome was classified according to the Cerebral Performance Category (CPC) to either good (CPC = 1–2) or poor (CPC = 3–5). Based on this outcome, the patients were assigned to one of two groups.

**Fig. 2:**
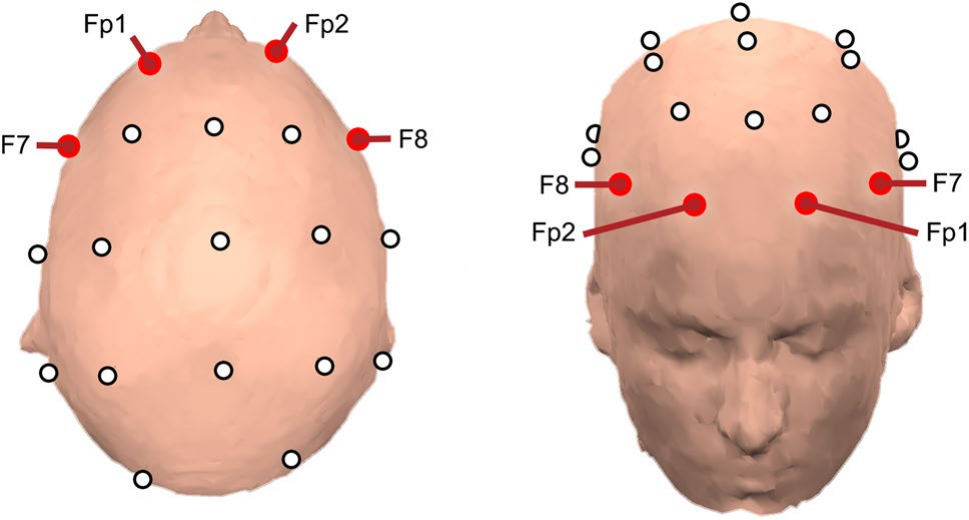
The location of electrodes for full 19-channel EEG cap and the four forehead channels used in the analysis

Slow wave activity was computationally defined by extracting the low-frequency power of the EEG. A 5-min signal sample was picked before each decrease in the drug infusion rate and at the end of the experiment. From the 5-min signal samples, a maximum of four 30-s artifact-free sequences were visually selected. Power spectral density (PSD) was calculated for these selected signal sequences using Welch’s averaged periodogram method [13] with 5-s Hamming window and 4.9-s overlap. An average over the maximum of four sequences was computed to represent a robust PSD estimate. Low-frequency power of the signal was then determined by summing the components below 1 Hz from the PSD estimates. Finally, average low-frequency power was calculated separately for the four forehead channels (Fp1, Fp2, F7, and F8) and all 19 channels (Fig. 2). These four forehead channels were chosen, as they are located in the hairless area and could thus be easily accessed with novel self-adhesive disposable electrodes. The EEG processing was carried out offline using the Matlab technical computing language (The MathWorks Inc., Natick, MA) version 2011b and the topographic plots were made with EEGLAB v13 [14].

Statistical comparison of the effect of infusion rate and group (independent variables) on the average low-frequency power of EEG was performed. The comparison was carried out separately for low-frequency power calculated from the four forehead channels and all 19 channels. Linear Mixed Model (LMM) with random intercept for subjects was used for analyzing repeatedly measured data. If infusion rate × group interaction was significant (P < 0.05), the infusion rate-wise comparisons between groups were performed. Statistical analysis was performed using SAS (version 9.3. SAS Institute Inc., Cary, NC, USA).

## Results

Figure 1 illustrates the slow wave activity in the four forehead channels and all 19 channels of the full EEG cap during the experiment in two individual patients: one with a good outcome and the other with a poor outcome. For the patient with a good outcome, high slow wave activity was seen at the beginning of the recording with high propofol infusion rates. The activity decreased as the infusion rate decreased. While this phenomenon was seen in all channels, the activity was emphasized in the frontal channels, which had higher average slow wave activity throughout the experiment compared to the global average calculated over the all 19 channels. For the patient with a poor outcome, slow wave activity at the beginning of the experiment was on a much lower level compared to that of the patient with a good outcome. Consequently, decreasing the propofol infusion rate had no substantial effect on the amount of slow wave activity. However, similarly to the patient with a good outcome, the slow wave activity of the patient with a poor outcome was also higher in the frontal channels than in the average of all the channels throughout the recording.

**Fig. 3:**
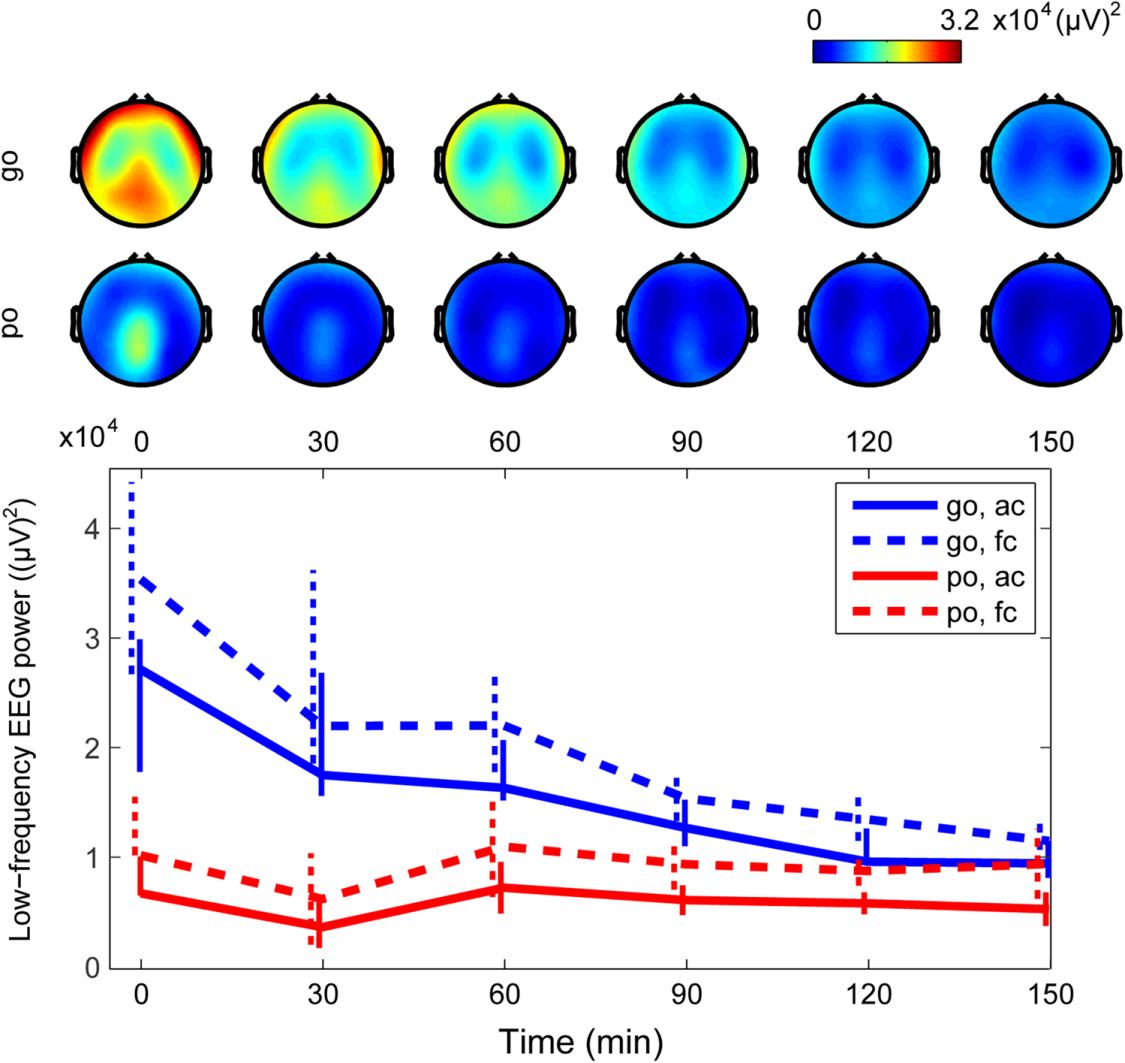
Slow wave activity of four forehead channels and all 19 channels of full EEG cap in the groups of good and poor neurological outcome. Above, topographic distribution of low-frequency (< 1 Hz) EEG power representing the slow wave activity at different phases of the experiment is given for patients with good outcome (go) and poor outcome (po). The values represent medians calculated over the groups. Below, the average low-frequency EEG power during the experiment in the four forehead channels (fc) and all 19 channels (ac) is presented for patients with good and poor neurological outcome. The trends are the medians calculated over the groups. The vertical lines represent the first and third quartiles of the data

The comparison of slow wave activity as captured by the four frontal electrodes and by all 19 channels of the full EEG cap are given in Fig. 3 for the groups of good (N = 6) and poor (N = 4) neurological outcome. Regardless of the group, the average slow wave activity calculated from the four forehead channels was on a somewhat higher level throughout the recording compared to the average calculated from all 19-channels. As with the example of the first patient in Fig. 1, slow wave activity was high in the good outcome group at high propofol infusion rates decreasing substantially towards the end of the experiment and infusion rate zero. Compared to the values at infusion rate 0 mg kg^−1^ h^−1^, the propofol-induced increase in the low-frequency power at the maximum infusion rate (4 mg kg^−1^ h^−1^) was 244 ± 91% (mean ± SD) in the four frontal channels. On the contrary, the patients with a poor outcome seemed to be incapable of producing propofol-induced frontal slow wave activity as their EEG low-frequency power in the four channels stayed low at all infusion rates being − 37 ± 88% at the maximum infusion rate compared to infusion rate 0 mg kg^−1^ h^−1^.

In the statistical analysis, the infusion rate did not have significant effect on the average low-frequency power calculated from the forehead channels (*P* = 0.32) or all the channels (*P* = 0.81) when the group information was not considered. Furthermore, the effect of group on the low-frequency EEG power was not significant regardless of whether it was calculated from the forehead channels (*P* = 0.42) or all the channels (*P* = 0.48) when the infusion rate information was not considered. However, a significant infusion rate × group interaction on low-frequency power was observed similarly with the forehead leads (*P* < 0.0001) as with the full EEG cap (*P* < 0.0001). The infusion rate-wise comparisons (see Table 1) showed statistically significant difference between the groups at infusion rate 4 mg kg^−1^ h^−1^ with forehead channels (*P* < 0.01) similar to all channels (*P* < 0.01).

**Table 1:** Low-frequency activity at different propofol infusion rates

Infusion rate (mg kg^−1^ h^−1^)	4		3		2		1		0.5		0	
	Median (range)	*P*	Median (range)	*P*	Median (range)	*P*	Median (range)	*P*	Median (range)	*P*	Median (range)	*P*
All channels												
Good outcome	2.71 (1.46–3.31)	< **0.01**	1.75 (1.37–3.16)	0.11	1.63 (1.30–2.40)	0.42	1.27 (0.32–1.60)	0.96	0.96 (0.47–1.44)	0.55	0.94 (0.35–1.32)	0.27
Poor outcome	0.70 (0.03–1.06)		0.40 (0.028-2.50)		0.72 (0.12–3.04)		0.61 (0.20–3.15)		0.58 (0.16–4.45)		0.53 (0.13–5.68)	
Forehead channels												
Good outcome	3.54 (2.26–6.34)	< **0.01**	2.20 (1.74–4.85)	0.09	2.20 (1.56–3.32)	0.57	1.54 (0.42–1.83)	1.00	1.35 (0.68–1.65)	0.65	1.15 (0.50–1.59)	0.24
Poor outcome	1.02 (0.04–1.55)		0.62 (0.04–3.03)		1.10 (0.25–4.60)		0.94 (0.23–3.51)		0.88 (0.20–4.90)		0.94 (0.15–6.97)	

Bold represent the significant values of *P* < 0.05

The units are 10^4^ (µV)^2^

## Discussion

This study shows that forehead electrodes sufficiently detect propofol-induced slow waves in the assessment of brain function after CA. In the experimental data from ten comatose post-CA patients, the performance of four forehead electrodes in capturing the slow wave activity differentiating the patients with good and poor neurological outcome was comparable to that of full 19-channel EEG cap. Since a low number of electrodes would make EEG-based approaches more feasible in the ICU, the finding offers potential in developing a clinically practical method for early detection of HIE.

The findings related to the reliability of forehead electrodes in capturing slow wave activity during the administration of anesthetics are supported by previous research. It has been shown that propofol-induced changes in the EEG are most prominently seen in the frontal montages [15]. While this “anteriorization” effect has been well described in alpha activity, whose power is totally lost in posterior areas during anesthesia [16], the same frontal dominance is seen in lower frequencies [17]. Purdon et al. [9] reported that relative increase in low-frequency EEG power (< 1 Hz) is seen broadly across almost the whole scalp. Our findings are in line with this emphasizing that, while propofol-induced increase in low-frequency EEG power is seen in all channels, the power values are above average in the frontal channels regardless of the propofol infusion rate. Hence, considering the challenges related to signal-to-noise ratio of EEG recordings, our findings may even suggest the superiority of frontal channels in this assessment task. However, larger dataset is needed to validate this suggestion.

With current data, the slow wave activity of patients with poor and good outcome was statistically significantly different only with highest propofol infusion rate (4 mg kg^−1^ h^−1^). While Fig. 3. suggests that larger dataset would be likely to produce significant difference also with lower infusion rates, it should be noted that the groups might substantially overlap on these. In other words, bringing out slow wave activity and this way detect HIE might require rather high propofol infusion rate or additional drug boluses. This should be investigated in future studies with larger dataset.

While the study shows potential for a technology that would automatically detect frontal slow wave activity from comatose CA patient providing prognostic information to the intensivists, several aspects related to signal analysis need to be considered before such technology would be reliable enough for clinical use. One of the challenges is related to the confirmation of the genuineness of the slow wave activity. Several other phenomena may increase the low-frequency power causing possible misinterpretation of the signal. Epileptic and periodic activity, for example, are quite frequently seen in post-CA patients. In a recent study, Backman et al. reported that 32% of the CA patients treated with target temperature management in the ICU suffer from electrographic status epilepticus [18]. A major part of these are non-convulsive showing no clinical signs [19]. Furthermore, periodic discharges often related to irreversible hypoxic brain damage [20] are a typical EEG finding in this patient group. Both epileptic activity and periodic discharges may affect the low-frequency power of EEG although not associated in any way to normal brain function like slow waves. The same applies to some of the non-neural signal sources such as ventilator and ECG artifacts. A reliable technology that automatically analyzes slow wave activity would need to remove these signal components that erroneously increase the low-frequency power of EEG. Considering the usage of only few forehead electrodes, the recognition and removal of erroneous signal components might be more challenging compared to when dealing with the recordings from full EEG cap.

The study has several limitations from which the most important ones are related to the size of the dataset and the clinical protocol. The small amount of patients included in this experimental pilot study prevents us from making any strong conclusions about the study results. The reliability of frontal electrodes in capturing propofol-induced slow wave activity should be confirmed in a wider study preferably including more than one study center to confirm the independence of the findings from ICU-specific practical issues. Furthermore, repeated or continuous EEG measurement should be applied to investigate how early after the CA slow waves can separate the patients with a good and with a poor outcome, and if the channels used have an impact on that. While the clinical protocol used in the current study including controlled exposure of the patient to different infusion rates of propofol is suitable in bringing out propofol-induced slow waves, it might not be suitable for clinical practice. Optimally, it should be possible to assess frontal slow waves during routine propofol administration used for the sedation of post-CA patients during temperature management treatment which should be considered in the clinical protocols of upcoming studies. In addition, the sedation of these patients might not be maintained with only propofol but include the co-administration of benzodiazepines and opiates, for example. The effect of these additional drugs on frontal slow waves should be investigated as well. To assess in more detail what is the sufficient effect-site concentration of propofol to produce enough slow wave activity to differentiate poor and good outcome groups, one could also apply target controlled infusion instead of fixed infusion rates in the study protocol.

Despite the limitations of the study, the findings provide an intriguing future view in the brain monitoring of post-CA patients. The quick progress made in EEG measurement systems, including the wireless and computational properties, have made it easier to apply online analysis even in a demanding clinical environment. Recently, a disposable forehead electrode set suitable for the ICU was launched and claimed to have excellent signal quality [21, 22]. These advances in the recording instruments make it possible for easy EEG data acquisition at the ICU and thus promote the development and improvement of algorithms for signal analysis providing diagnostic and prognostic information. Considering this, it is easy to see in the future EEG measurement with automatic analysis as a part of the routine monitoring of post-CA ICU patients helping the doctors in decision making.
